# Implementation of a Personalized Medicine Approach in Patients With Type 2 Diabetes Mellitus Receiving Multiple Daily Insulin Injections (POMA Project): Protocol for a Before-and-After Intervention Study

**DOI:** 10.2196/85375

**Published:** 2026-02-24

**Authors:** Rosa Giné-Balcells, Bogdan Vlacho, Olga Carmen Vidal-Pérez, Albert Lecube, Josep Franch-Nadal, Dídac Mauricio, Àngels Molló-Iniesta, Marta Hernández, POMA Study Group

**Affiliations:** 1 Primary Health Care Center Tàrrega, Gerència d'Atenció Primària Lleida, Institut Català de la Salut, Lleida, Spain; 2 CIBER de Diabetes y Enfermedades Metabólicas Asociadas, Instituto de Salud Carlos III, Barcelona, Spain; 3 DAP-Cat group, Unitat de Suport a la Recerca Barcelona, Fundació Institut Universitari d'Investigació en Atenció Primària Jordi Gol i Gurina (IDIAPJGol), Barcelona, Spain; 4 Department of Endocrinology and Nutrition, University Hospital Arnau de Vilanova, Avda. Alcalde Rovira Roure, 80, Lleida, 25198, Spain, +34 973705183; 5 Endocrinology Department, Vall d'Hebron University Hospital, Autonomous University Barcelona, Barcelona, Spain; 6 Diabetes and Metabolism Research Group, Vall d’Hebron Research Institute (VHIR), Barcelona, Spain; 7 Primary Health Care Center, Gerència d'Àmbit d'Atenció Primària Barcelona Ciutat, Institut Català de la Salut, Barcelona, Spain; 8 Research Group in Endocrinology, Diabetes and Nutrition, Institut de Recerca Sant Pau, Barcelona, Spain; 9 Department of Endocrinology and Nutrition, Hospital de la Santa Creu i Sant Pau, Barcelona, Spain; 10 Faculty of Medicine, University of Vic - Central University of Catalonia (UVIC/UCC), Vic, Spain; 11 Institut de Recerca Biomèdica de Lleida Fundació Dr. Pifarré, University of Lleida, Lleida, Spain; 12 Primary Health Care Center, Gerència d'Atenció Primària Lleida, Institut Català de la Salut, Lleida, Spain

**Keywords:** type 2 diabetes mellitus, multiple insulin doses, continuous glucose monitoring, precision medicine, anti-GAD65 autoantibody, C-peptide

## Abstract

**Background:**

The management of type 2 diabetes mellitus (T2DM) remains a complex clinical challenge, particularly for patients requiring multiple daily insulin injections (MDI). Advances in precision medicine and continuous glucose monitoring (CGM) have created opportunities to personalize treatment and potentially reduce the therapeutic burden on people with T2DM. Assessing β cell function and autoimmunity could help identify patients with T2DM eligible for simplified regimens without compromising glycemic control.

**Objective:**

The aim of this study is to test a simple personalized medicine protocol in routine clinical practice for people with T2DM treated with MDI. The intervention is based on evaluating C-peptide and glutamic acid decarboxylase autoantibody status with the goal of improving diagnostic accuracy and optimizing treatment.

**Methods:**

This is a pragmatic before-and-after intervention study involving people with T2DM currently receiving MDI across primary care centers and a referral hospital in the Lleida health care region in Catalonia (Spain). Eligible participants will undergo clinical and laboratory assessment, including C-peptide and glutamic acid decarboxylase autoantibody testing, and wear a CGM device. On the basis of a predefined algorithm, patients may either continue or discontinue prandial insulin. The primary outcome is the proportion of patients in whom prandial insulin is discontinued and remains discontinued over 6 months. Secondary outcomes include changes in hemoglobin A_1c_, CGM metric variables, quality of life, adherence, and treatment satisfaction.

**Results:**

Recruitment was completed on March 31, 2025. The follow-up phase is ongoing and expected to conclude by September 30, 2025. Data analysis will begin thereafter.

**Conclusions:**

This study will evaluate the feasibility and impact of implementing a personalized therapeutic approach for persons with T2DM receiving MDI in real-world clinical settings. If effective, this strategy could contribute to safer, simpler, and more individualized diabetes care.

## Introduction

### Type 2 Diabetes Mellitus and Continuous Glucose Monitoring

Diabetes mellitus (DM) is considered a global pandemic with increasing incidence and prevalence and is expected to affect 853 million people worldwide by 2050 [1]. Type 2 DM (T2DM) is a chronic and progressive disease characterized by peripheral insulin resistance and a decline in β cell function [2,3]. The degree of insulin resistance and secretion, the rate of deterioration, the associated metabolic phenotype, the response to treatment, and the occurrence of chronic complications vary across individuals [4].

Although T2DM accounts for over 90% of diabetes cases worldwide [1], a significant proportion of patients diagnosed with T2DM may actually have other forms of diabetes. Among these, latent autoimmune diabetes in adults (LADA) is noteworthy. LADA, a slowly progressive form of autoimmune diabetes, is characterized by the presence of pancreatic autoantibodies and a faster progression to insulin dependence than is typically observed in T2DM [5]. It is often misdiagnosed due to reliance on the clinical phenotype alone [6]. The prevalence of LADA varies by population and geography, ranging from 3% to 12% of all persons with DM [7]. Diagnosis requires detection of at least one pancreatic autoantibody, with the most common being glutamic acid decarboxylase autoantibody (GADAb) [8].

Managing T2DM remains a significant clinical challenge as its progressive nature often increases treatment complexity over time. While approximately 10% of patients eventually require complex insulin regimens [9], newer pharmacological agents and individualized treatment strategies may allow for treatment simplification, reduction of hypoglycemia risk, and improvements in quality of life when aligned with personalized glycemic targets [10-13]. C-peptide measurement can be used to assess endogenous insulin production and serve as a proxy for residual β cell function. This information may help determine whether prandial insulin is necessary or could be safely discontinued in favor of alternative therapies [14].

A randomized controlled trial by Rosenstock et al [15] demonstrated that, in people with T2DM, discontinuing prandial insulin and initiating a once-weekly glucagonlike peptide-1 receptor agonist led to improved glycemic control approaching normoglycemia, reduced hypoglycemia, decreased body weight, and improved quality of life. Similarly, the BEYOND trial by Giugliano et al [16] evaluated switching from basal bolus insulin regimens to a glucagonlike peptide-1 receptor agonist or a sodium-glucose cotransporter type 2 inhibitor. Comparable glycemic control was achieved with lower total insulin doses, fewer daily injections, and reduced hypoglycemia, along with improved weight management and patient satisfaction. A comprehensive review by Giugliano et al [17] including these and other studies confirmed that insulin therapy can be simplified in persons with T2DM without compromising glycemic control while reducing treatment complexity, hypoglycemia risk, and polypharmacy.

Continuous glucose monitoring (CGM) has been evaluated in both randomized trials and real-world studies involving people with T2DM treated with multiple daily insulin injections (MDI), basal insulin, or noninsulin regimens [18,19]. In Catalonia (northeast Spain), the public implementation of CGM for people with T2DM on MDI was approved in November 2023. This required collaboration and coordination between different care providers, constituting an opportunity to develop and implement this protocol in routine clinical practice [20].

This project proposes implementing a precision medicine algorithm for persons with T2DM treated with MDI in real-world clinical practice. This algorithm incorporates assessment of pancreatic autoimmunity and residual β cell function (pancreatic reserve) into the decision-making workup.

### Hypothesis and Objectives

We hypothesize that approximately 25% of people with T2DM on MDI have sufficient β cell secretory capacity to safely discontinue prandial insulin. In these cases, treatment could be optimized by introducing agents with proven cardio-renal or weight-lowering benefits according to current clinical guidelines.

The main objective is to evaluate a simple personalized medicine protocol in routine clinical practice for people with T2DM on MDI. The protocol, based on clinical and analytical parameters, aims to improve diagnostic accuracy and potentially simplify treatment in this population.

Secondary objectives include describing patient characteristics; conducting detailed phenotyping of pancreatic function and autoimmunity status; and evaluating the impact of the intervention on glycemic control, treatment adherence, and quality of life.

## Methods

This paper reports a study protocol developed in accordance with the SPIRIT (Standard Protocol Items: Recommendations for Interventional Trials) guidelines.

### Study Design and Settings

We designed a nonrandomized pragmatic intervention study targeting people diagnosed with T2DM who were treated with MDI. This before-and-after intervention was designed to assess the real-world applicability of a precision medicine algorithm. Rather than using a control group, this study uses each participant as their own control, comparing clinical outcomes before and after the intervention. The intervention was adapted to be integrated into routine clinical workflows across both primary and specialized care settings.

This study is being conducted within 21 primary health care centers of the Catalan Health Institute (Institut Català de la Salut) in the Lleida health care region (Catalonia, Spain; Multimedia Appendix 1) together with the Department of Endocrinology and Nutrition, University Hospital Arnau de Vilanova, as the reference tertiary center.

### Eligibility Criteria

We included adults aged ≥18 years with a confirmed diagnosis of T2DM. To be enrolled in the study, participants must be on an MDI regimen, defined as receiving at least 2 doses of prandial insulin per day (either regular insulin or a rapid-acting insulin analogue) administered separately or as part of a fixed-dose combination.

We excluded individuals with a known diagnosis of diabetes other than T2DM, pregnant women, and those with severe cognitive impairment or any condition that prevented the use of CGM. We also excluded participants with current drug or alcohol abuse, those receiving systemic glucocorticoids for acute conditions at the time of inclusion, and individuals undergoing temporary insulin therapy expected to last less than 6 months.

### Recruitment and Sampling Strategy

The participant selection sampling strategy was a convenient approach based on the availability and willingness to participate of the patients who fulfilled the study criteria [21].

Participating centers were selected based on geographic distribution and availability of primary health care teams. Teams received virtual training sessions. Sessions included information related to the background, objectives, protocol procedures, and data collection methods before participant recruitment. The study recruitment period lasted 6 months.

### Study Procedures and Timeline

**Figure 1.**
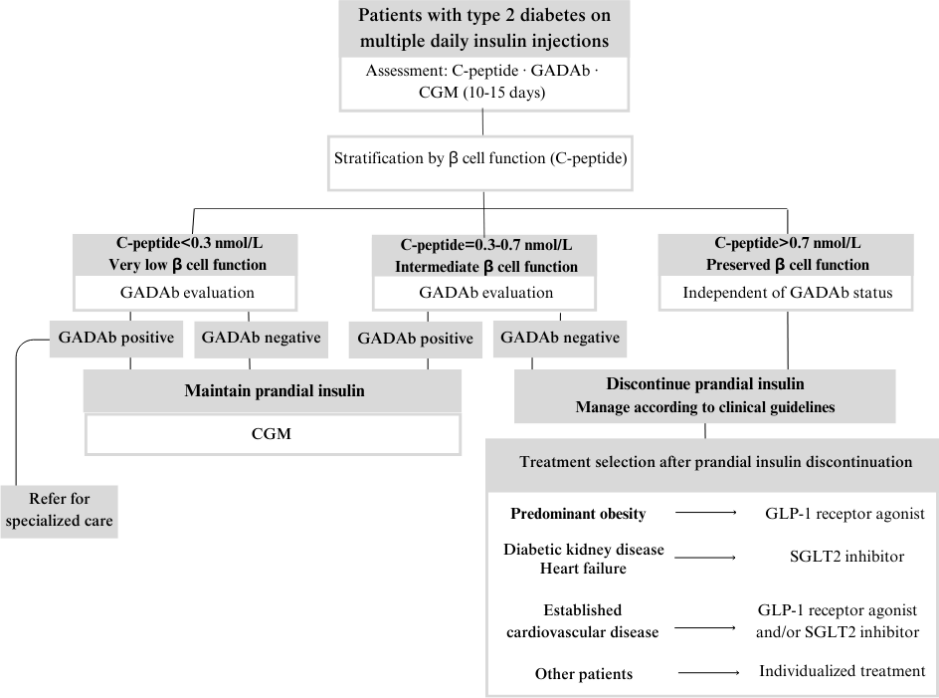
Therapeutic intervention algorithm. CGM: continuous glucose monitoring; GADAb: glutamic acid decarboxylase autoantibody; GLP-1: glucagonlike peptide-1; SGLT2: sodium-glucose cotransporter type 2.

Potential participants were identified by local study physicians and nurses using the primary care eCAP (Catalan Health Institute) electronic clinical record software. Each participant underwent 4 study visits. Initially, a preselection visit was conducted through electronic health record review without direct patient contact. Eligible individuals were then contacted and provided with detailed study information. Those who agreed to participate were invited to the inclusion visit (visit 0), during which written informed consent was obtained by research staff. This visit involved a clinical interview, a physical examination, laboratory testing (including C-peptide and GADAb measurements), and the placement of a CGM device. All participants used CGM for 10 to 15 days prior to the intervention. Two weeks later, the first follow-up visit (visit 1) was conducted. At this visit, therapeutic decisions were made based on C-peptide and GADAb results, and treatment was adjusted according to the predefined therapeutic intervention algorithm (Figure 1).

The second follow-up and final in-person study visit (visit 2) took place at month 6, during which outcomes were assessed. Glycemic control was evaluated through hemoglobin A_1c_ (HbA_1c_) testing, and other clinically relevant follow-up variables were collected. Throughout the study period, participants received additional follow-up and support as needed via telephone or virtual consultations from their primary health care team.

**Figure 2.**
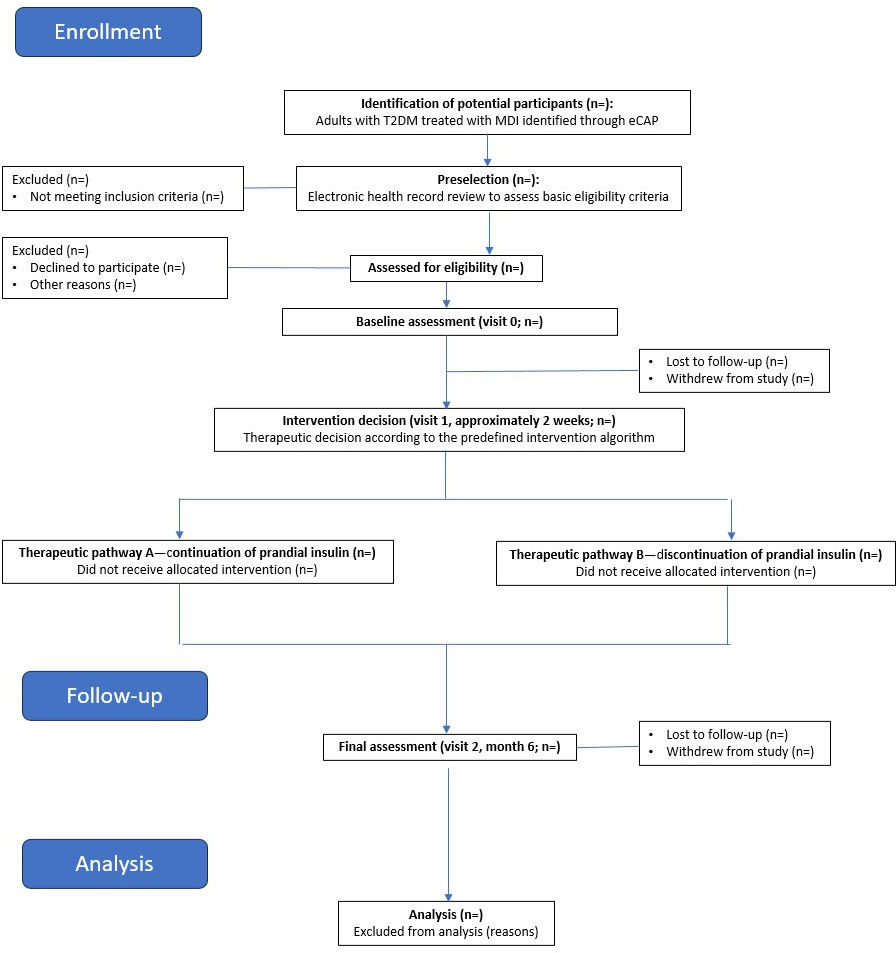
Study flowchart. eCAP: electronic clinical record software of primary care (Catalonia); MDI: multiple daily insulin injection; T2DM: type 2 diabetes mellitus.

Figure 2 illustrates the planned participant flow and study procedures across the different study visits.

### Data Collection and Management

#### Overview

Data for this study are collected from electronic medical records and patient-reported questionnaires during study visits. Clinical and demographic information, including age, sex, educational level, BMI, diabetes duration, comorbidities, complications, and current pharmacological treatment, is obtained from the eCAP (electronic medical record) software and supplemented by medical interviews. Laboratory variables, including C-peptide, GADAb, HbA_1c_, and estimated glomerular filtration rate, are extracted from recent laboratory reports or requested when unavailable. CGM data, including metrics such as time in range, hypoglycemia episodes, and glucose variability, are downloaded directly from the devices used by participants throughout the study. Patient-reported outcomes, including quality of life, treatment satisfaction, medication adherence, and physical activity, are assessed using validated questionnaires. All study variables and corresponding time points are summarized in Table 1. In addition, detailed information on the study variables, including their definitions and data sources, is provided in Multimedia Appendix 2.

**Table 1.** Study timeline, variables, and procedures.

Data collected	Evaluation time point			
	Preselection	Visit 0—inclusion	Visit 1—intervention (2 weeks)	Visit 2—final follow-up (6 months)
Inclusion and exclusion criteria	✓	✓		
Sociodemographic data^a^		✓		
Anthropometric data^b^		✓		✓
Clinical data^c^		✓		✓
CGM^d^ data			✓	✓
Laboratory data^e^		✓		✓
Smoking status		✓		
Pharmacological treatment^f^		✓		✓
Severe hypoglycemia^g^		✓		✓
Questionnaires^h^		✓		✓

a Age, gender, primary care center, current occupational status, level of education, number of children, date of menopause, and whether the participant is the sole caregiver of another person.

b Weight, height, BMI, and abdominal circumference.

c Arterial hypertension, dyslipidemia, micro- and macrovascular complications, heart failure, and renal disease of any etiology (estimated glomerular filtration rate <60 mL per minute per 1.73 m2).

d CGM: continuous glucose monitoring.

e Inclusion visit: hemoglobin A_1c_ (HbA_1c_), glutamic acid decarboxylase autoantibody, glycemia, and C-peptide; final visit: HbA_1c_.

f Prescription and dosage of diabetes drugs.

g Inclusion visit: last 12 months; final visit: 6 months of study.

h International Physical Activity Questionnaire, Diabetes Treatment Satisfaction Questionnaire–Status Version, Diabetes Treatment Satisfaction Questionnaire–Change Version (final visit only), Adherence to Refills and Medications Scale–Spanish Version, and Spanish version of the Diabetes Quality of Life Questionnaire.

All data are systematically recorded using an electronic case report form (eCRF) specifically developed for this study. Local investigators at each participating center are responsible for ensuring accurate, complete, and timely entry of study data into the eCRF. We perform quality control procedures to verify informed consent and participant eligibility prior to data entry. In addition, the coordinating research team regularly monitors the eCRF data entries to ensure high data quality and compliance with good clinical practice standards throughout the study.

#### Questionnaires

Several validated questionnaires are used to assess patient-reported outcomes related to quality of life, treatment satisfaction, medication adherence, and physical activity. These questionnaires are administered at both the baseline and final study visits (6 months), as outlined in the study timeline (Table 1).

A detailed description of each questionnaire, including constructs assessed, number of items, scoring, and references, is provided in Table 2.

**Table 2.** Summary of patient-reported outcome questionnaires used in this study.

Questionnaire	Construct measured	Description and domains	Number of items	Response scale	Scoring and interpretation	Time points	Reference
ESDQoL^a^	Diabetes-related quality of life	Assesses multiple domains of diabetes-specific quality of life, with domain impact weighted by patient-reported importance	43	5-point Likert scale	Weighted average score; higher scores indicate poorer quality of life	Baseline and 6 months	[22]
DTSQs^b^	Diabetes treatment satisfaction	Measures satisfaction with current diabetes treatment; includes separate items on perceived frequency of hyperglycemia and hypoglycemia	8	7-point Likert scale (0 to 6)	Sum of 6 items (range 0 to 36); higher scores indicate greater satisfaction; 2 items analyzed separately	Baseline and 6 months	[23]
DTSQc^c^	Change in treatment satisfaction	Assesses perceived change in satisfaction compared with previous treatment	8	7-point Likert scale (–3 to +3)	Positive scores indicate improvement; negative scores indicate deterioration	6 months	[23]
ARMS-e^d^	Medication adherence	Evaluates adherence behaviors and barriers related to medication use	12	4-point Likert scale (“never” to “always”)	Lower total scores indicate better adherence	Baseline and 6 months	[24]
IPAQ^e^	Physical activity	Assesses physical activity in the previous 7 days, including walking, moderate and vigorous activity, and sedentary behavior	7	Frequency (d per wk) and duration (min per d)	Physical activity expressed as MET^f^ min per week; higher values indicate higher activity levels	Baseline and 6 months	[25]

a ESDQoL: Spanish Diabetes Quality of Life Questionnaire.

b DTSQs: Diabetes Treatment Satisfaction Questionnaire–Status Version.

c DTSQc: Diabetes Treatment Satisfaction Questionnaire–Change Version.

d ARMS-e: Adherence to Refills and Medications Scale–Spanish Version.

e IPAQ: International Physical Activity Questionnaire–Short Form (Spanish version).

f MET: metabolic equivalent of task.

### Intervention Algorithm

Therapeutic decisions in the study are guided by an algorithm based on C-peptide and GADAb levels (Figure 1). Patients with a C-peptide level below 0.3 nmol/L and positive GADAb are considered suspected type 1 diabetes cases; they continue MDI and CGM and are referred for specialized care. Those with similarly low C-peptide levels but negative GADAb also continue MDI and CGM with no changes in treatment.

For patients with intermediate C-peptide values (0.3‐0.7 nmol/L), the approach varies based on GADAb status. If GADAb is positive, treatment remains unchanged with continued MDI and CGM. However, if GADAb is negative, clinicians consider discontinuing prandial insulin and introducing noninsulin hypoglycemic agents as appropriate.

Participants with C-peptide levels above 0.7 nmol/L regardless of GADAb status are also considered for discontinuation of prandial insulin and initiation of noninsulin therapies. In individuals who discontinue prandial insulin, CGM use is typically discontinued as well as they do not meet the criteria for public funding [26].

### Study Outcomes

We defined the primary study outcome as the proportion of participants in whom prandial insulin is successfully discontinued after the application of the therapeutic algorithm and who remain without prandial insulin at the final visit (6 months).

Secondary outcomes encompass several domains. Glycemic control following therapeutic optimization is evaluated through changes in HbA_1c_ and CGM-derived metrics, including time in range and time below range. Patient-reported outcomes are also assessed by comparing changes from baseline to 6 months after the intervention, including changes in quality of life (using the Diabetes Quality of Life Questionnaire), treatment satisfaction (using the Diabetes Treatment Satisfaction Questionnaire, both status and change versions), medication adherence (measured using the Adherence to Refills and Medications Scale), and physical activity levels (evaluated using the Spanish version of the International Physical Activity Questionnaire).

Safety outcomes include the number of severe hypoglycemic episodes at the final visit 6 months after the intervention compared with the 12-month period before enrollment. The study also examines the predictive value of CGM metrics by analyzing their association with pancreatic reserve and insulin resistance considering both continuous and categorical variables.

Finally, among patients who continue prandial insulin and CGM use for 6 months, the study assesses the impact of CGM on glycemic control (HbA_1c_ and CGM metrics), as well as changes in quality of life, treatment satisfaction, and medication adherence.

### Sample Size and Statistical Analysis

At the time of the study planning, data from the Lleida health care region had identified 1388 people with T2DM treated with MDI. On the basis of prior clinical experience, approximately 25% of these individuals may have sufficient residual β cell function to discontinue prandial insulin. To estimate this proportion with a 95% CI and a margin of error of –5% to +5%, a sample size of 239 participants was estimated.

The study population will be characterized at baseline with respect to age, BMI, duration of diabetes, presence of chronic diabetes-related complications (microvascular and macrovascular), and cardiovascular risk factors. In addition, in-depth phenotyping will be performed using the homeostatic model assessment of β cell function and homeostatic model assessment of insulin resistance indexes [27] and GADAb measurements.

For the primary outcome, the proportion of participants who discontinue prandial insulin after the application of the diagnostic algorithm and sustain this change over a 6-month follow-up period will be calculated, along with its corresponding 95% CI.

Secondary outcomes will include measures of glycemic control, patient-reported outcomes, and safety outcomes. Glycemic control will be assessed using HbA_1c_ and CGM-derived metrics, including time in range (70‐180 mg/dL), time below range, time above range, mean glucose, and glycemic variability indexes. Patient-reported outcomes will include validated scores for treatment satisfaction, adherence, and quality of life analyzed according to each instrument’s scoring manual. Safety outcomes will include the incidence of severe hypoglycemia.

To evaluate changes over time (eg, from baseline to follow-up visits), paired 2-tailed *t* tests or Wilcoxon signed-rank tests will be used depending on data distribution. Between-group comparisons (eg, discontinuation vs continuation of prandial insulin) will be conducted using Student *t* tests or Mann-Whitney *U* tests as appropriate, as well as using linear mixed-effects models to account for within-subject correlation.

For categorical secondary outcomes, including severe hypoglycemia, comparisons between groups will be performed using chi-square or Fisher exact tests as appropriate. For outcomes measured repeatedly across follow-up visits, analyses will be conducted using linear mixed-effects models.

CGM variables will also be examined as potential predictors of pancreatic reserve and insulin resistance. Depending on whether outcomes are continuous or categorical, linear or logistic regression models will be applied. Results will be expressed as regression coefficients or odds ratios with corresponding 95% CIs.

Loss to follow-up will be quantified and reported as the proportion of participants without outcome data at each follow-up time point. When available, reasons for loss to follow-up will be documented. Baseline characteristics of participants with complete follow-up and those lost to follow-up will be compared to assess potential differential attrition. A complete-case analysis will be used as the primary analytical approach when the proportion of missing data is low, and missingness will be considered compatible with a missing completely at random mechanism. To account for potentially informative missingness, multiple imputation by chained equations will be performed under a missing-at-random assumption, including all variables used in the main analyses and auxiliary variables related to missingness.

All statistical analyses will be performed using the R software (version 4.1.2; R Foundation for Statistical Computing), with a 2-sided *P* value of <.05 considered statistically significant.

### Ethical Considerations

#### Overview

This study will be conducted in accordance with the Declaration of Helsinki and the principles of good clinical practice. The study protocol received approval from the ethics committee of Institut Universitari d’Investigació en Atenció Primària Jordi Gol i Gurina, the research institute for primary care in Catalonia. The approved protocol is registered under reference 23/193-EOm. Participants did not receive any financial or material compensation for taking part in the study.

The trial is registered at ClinicalTrials.gov with the identifier NCT06148376.

#### Informed Consent

All participants received verbal and written information about the study and provided signed informed consent before enrollment. When participants had limited capacity to consent, their legal representatives may provide authorization.

#### Data Protection and Confidentiality

All personal data will be pseudonymized and processed in compliance with the General Data Protection Regulation (2016/679) and Spanish Organic Law 3/2018 on the Protection of Personal Data and Guarantee of Digital Rights. Individual patient information will be encrypted to ensure anonymity. Only authorized researchers and study monitors will have access to the data.

### Publication Commitment

The results of the study will be disseminated regardless of the outcome and will be submitted for peer-reviewed publication. Participant confidentiality will be maintained in all presentations and publications.

## Results

Recruitment for the study began in January 2024 and was completed on March 31, 2025.

As of August 31, 2025, the study is in its final follow-up phase, scheduled to conclude on September 30, 2025. Formal data analysis will begin after completion of follow-up and final data collection.

## Discussion

### Expected Findings

According to data from the Catalan Agency for Health Quality and Assessment and the eCAP electronic medical record system, the Lleida health care region has approximately 27,769 individuals diagnosed with T2DM, representing 8.95% of the adult population. Among these patients, 5% are currently treated with MDI.

This study aims to improve the available evidence in precision medicine for T2DM, specifically targeting patients treated with MDI. Using the simple precision medicine algorithm (Figure 1) proposed in this study based on assessment of β cell function (C-peptide) and autoimmunity (GADAb), we will evaluate whether prandial insulin can be safely removed or replaced by another treatment in real-world clinical practice.

The study’s main outcome is the proportion of patients in whom prandial insulin is successfully discontinued and remains unnecessary at 6 months. Secondary outcomes include clinical measures such as HbA_1c_ levels; CGM-derived metrics related to insulin resistance and pancreatic function; incidence of severe hypoglycemia; and several patient-reported outcomes—including quality of life, treatment satisfaction, medication adherence, and physical activity. Among patients who continue prandial insulin and CGM, the study also examines the added value of CGM use on both metabolic control and patient experience.

The algorithm is implemented by a multidisciplinary research team—including general practitioners, endocrinologists, and nurses—who integrate biomarker results with patient-specific clinical characteristics to guide medical decision-making. The strategy proposed by this study is intended for inclusion in clinical practice. If successful, this approach could be adopted by health care professionals managing T2DM to achieve simpler, safer, and more effective treatment strategies, particularly those that offer cardiovascular, renal, and weight management benefits.

This personalized strategy aligns with a broader innovation trend in diabetes care. Similar to the study by Dennis et al [28], which developed and validated a predictive model to individualize glucose-lowering therapies in T2DM, our project aims to move beyond conventional treatment algorithms by incorporating patient-specific pathophysiological characteristics.

Adopting a simple, precision-based approach to T2DM represents both a challenge and an opportunity. It can foster improved coordination between primary and specialized diabetes care teams and enhance understanding of diabetes heterogeneity. This study exemplifies how precision strategies can be tested and implemented in standard care settings. By enabling more individualized treatment decisions, the intervention may reduce overtreatment, minimize hypoglycemia, enhance quality of life, empower patients, and strengthen the use of digital health tools and telemedicine in routine care.

### Limitations

This study has several limitations. First, as with many observational studies involving voluntary participation, there is potential selection bias. Individuals who choose to participate may differ systematically from those who do not, for example, by being more motivated, having greater health literacy, or exhibiting more stable diabetes management. Such characteristics could affect adherence, clinical outcomes, and openness to treatment simplification, thereby potentially limiting the generalizability of the findings [21].

Second, although the study includes all persons with a diagnosis of T2DM treated with MDI within the participating centers, recruitment depended on the initiative of local primary care teams, which may introduce variability in enrollment rates and patient selection. Third, the intervention algorithm offers flexibility in its clinical application, potentially leading to deviations from the proposed protocol. However, given the pragmatic nature of the study, this flexibility is considered necessary to ensure patient safety and adapt the intervention to the heterogeneous clinical profiles of T2DM, a condition influenced by multiple factors affecting treatment response. Lastly, the duration of diabetes may affect the reliability of autoantibody testing. In long-standing cases, GADAb titers may decline over time, leading to false-negative results. Although longitudinal data are limited, up to 40% of GADAb-positive patients may become seronegative 10 years after diagnosis [29]. Despite this, GADAb was selected over other autoantibodies due to its higher prevalence and persistence in patients with LADA [5].

In summary, these limitations—including the use of convenience-based recruitment, potential selection bias, variability in recruitment across centers, and the pragmatic flexibility of the intervention algorithm—should be considered when interpreting the findings. These factors may limit causal inference and applicability, but they are consistent with the study’s feasibility-focused, real-world nature.

### Conclusions

More accurate phenotyping of persons with T2DM on MDI may improve diagnostic accuracy and enable treatment optimization by identifying candidates for prandial insulin withdrawal or for the introduction of agents with better safety profiles and added cardiovascular benefits [30].

Simplifying therapeutic strategies in T2DM management can be effective provided that personalized care is prioritized, patient safety is preserved, and glycemic control is maintained.

Furthermore, implementing CGM represents both a challenge and an opportunity to enhance diabetes care across primary care and endocrinology services. Strengthening collaboration across care levels and promoting the use of digital health tools may empower patients and improve the management of this highly prevalent and impactful chronic disease.

## Supplementary material

10.2196/85375 Multimedia Appendix 1 List of study participating centers.

10.2196/85375 Multimedia Appendix 2 Study variables.

10.2196/85375 Checklist 1 SPIRIT checklist.

## Abbreviations

CGM: continuous glucose monitoring
DM: diabetes mellitus
eCRF: electronic case report form
GADAb: glutamic acid decarboxylase autoantibody
HbA_1c_: hemoglobin A_1c_
LADA: latent autoimmune diabetes in adults
MDI: multiple daily insulin injection
SPIRIT: Standard Protocol Items: Recommendations for Interventional Trials
T2DM: type 2 diabetes mellitus

## References

[1] (2025). IDF diabetes atlas. International Diabetes Federation.

[2] (1995). U.K. prospective diabetes study 16. Overview of 6 years’ therapy of type II diabetes: a progressive disease. U.K. prospective diabetes study group. Diabetes.

[3] ElSayed NA, McCoy RG, Aleppo G (2025). Diagnosis and classification of diabetes: standards of care in diabetes-2025. Diabetes Care.

[4] Redondo MJ, Hagopian WA, Oram R (2020). The clinical consequences of heterogeneity within and between different diabetes types. Diabetologia.

[5] Laugesen E, Østergaard JA, Leslie RDG, Danish Diabetes Academy Workshop and Workshop Speakers (2015). Latent autoimmune diabetes of the adult: current knowledge and uncertainty. Diabet Med.

[6] Hernández M, Mauricio D (2021). Latent autoimmune diabetes in adults: a review of clinically relevant issues. Adv Exp Med Biol.

[7] Vich-Pérez P, Abánades-Herranz JC, Mora-Navarro G (2023). Development and validation of a clinical score for identifying patients with high risk of latent autoimmune adult diabetes (LADA): the LADA primary care-protocol study. PLoS ONE.

[8] Pieralice S, Pozzilli P (2018). Latent autoimmune diabetes in adults: a review on clinical implications and management. Diabetes Metab J.

[9] Vinagre I, Mata-Cases M, Hermosilla E (2012). Control of glycemia and cardiovascular risk factors in patients with type 2 diabetes in primary care in Catalonia (Spain). Diabetes Care.

[10] Artasensi A, Pedretti A, Vistoli G, Fumagalli L (2020). Type 2 diabetes mellitus: a review of multi-target drugs. Molecules.

[11] Jude EB, Malecki MT, Gomez Huelgas R (2022). Expert panel guidance and narrative review of treatment simplification of complex insulin regimens to improve outcomes in type 2 diabetes. Diabetes Ther.

[12] Mehta R, Billings LK, Liebl A, Vilsbøll T (2022). Transitioning from basal-bolus or premix insulin therapy to a combination of basal insulin and glucagon-like peptide-1 receptor agonist in people with type 2 diabetes. Diabet Med.

[13] Davies MJ, Aroda VR, Collins BS (2022). Management of hyperglycaemia in type 2 diabetes, 2022. A consensus report by the American Diabetes Association (ADA) and the European Association for the Study of Diabetes (EASD). Diabetologia.

[14] Maddaloni E, Bolli GB, Frier BM (2022). C-peptide determination in the diagnosis of type of diabetes and its management: a clinical perspective. Diabetes Obes Metab.

[15] Rosenstock J, Nino A, Soffer J (2020). Impact of a weekly glucagon-like peptide 1 receptor agonist, albiglutide, on glycemic control and on reducing prandial insulin use in type 2 diabetes inadequately controlled on multiple insulin therapy: a randomized trial. Diabetes Care.

[16] Giugliano D, Longo M, Caruso P (2021). Feasibility of simplification from a basal-bolus insulin regimen to a fixed-ratio formulation of basal insulin plus a GLP-1RA or to basal insulin plus an SGLT2 inhibitor: BEYOND, a randomized, pragmatic trial. Diabetes Care.

[17] Giugliano D, Scappaticcio L, Longo M (2021). Simplification of complex insulin therapy: a story of dogma and therapeutic resignation. Diabetes Res Clin Pract.

[18] American Diabetes Association Professional Practice Committee (2025). 7. Diabetes technology: standards of care in diabetes-2025. Diabetes Care.

[19] Ajjan RA, Battelino T, Cos X (2024). Continuous glucose monitoring for the routine care of type 2 diabetes mellitus. Nat Rev Endocrinol.

[20] Model organitzatiu per a la prestació dels sistemes de monitoratge continu de glucosa a pacients amb diabetis mellitus de tipus 2 al SISCAT. CatSalut: Servei Català de la Salut.

[21] Convenience sampling method: how and when to use it?. Qualtrics.

[22] Millan M (2002). Quality-of-life questionnaire designed for diabetes mellitus (EsDQOL). Aten Primaria.

[23] Bradley C, Bradley C (1994). Handbook of Psychology and Diabetes: A Guide to Psychological Measurement in Diabetes Research and Practice.

[24] Kripalani S, Risser J, Gatti ME, Jacobson TA (2009). Development and evaluation of the Adherence to Refills and Medications Scale (ARMS) among low-literacy patients with chronic disease. Value Health.

[25] Craig CL, Marshall AL, Sjöström M (2003). International physical activity questionnaire: 12-country reliability and validity. Med Sci Sports Exerc.

[26] (2022). Resolución de 7 de abril de 2022, de la dirección general de cartera común de servicios del sistema nacional de salud y farmacia, por la que se hace público el acuerdo de la comisión de prestaciones, aseguramiento y financiación de 2 de marzo de 2022 sobre sistema de monitorización de glucosa para pacientes con diabetes mellitus tipo 2 en la cartera común de servicios del sistema nacional de salud. https://www.sanidad.gob.es/ca/profesionales/prestacionesSanitarias/CarteraDeServicios/ContenidoCS/docs/Resoluc_DG__DM2_DEF.pdf.

[27] Diabetes trials unit: HOMA calculator. University of Oxford.

[28] Dennis JM, Young KG, Cardoso P (2025). A five-drug class model using routinely available clinical features to optimise prescribing in type 2 diabetes: a prediction model development and validation study. The Lancet.

[29] Tuomi T, Santoro N, Caprio S, Cai M, Weng J, Groop L (2014). The many faces of diabetes: a disease with increasing heterogeneity. The Lancet.

[30] Fritsche A, Heni M, Peter A (2022). Considering insulin secretory capacity as measured by a fasting C-peptide/glucose ratio in selecting glucose-lowering medications. Exp Clin Endocrinol Diabetes.

