# Correction: Implementation of a Personalized Medicine Approach in Patients With Type 2 Diabetes Mellitus Receiving Multiple Daily Insulin Injections (POMA Project): Protocol for a Before-and-After Intervention Study

**DOI:** 10.2196/95267

**Published:** 2026-04-13

**Authors:** Rosa Giné-Balcells, Bogdan Vlacho, Olga Carmen Vidal-Pérez, Albert Lecube, Josep Franch-Nadal, Dídac Mauricio, Àngels Molló-Iniesta, Marta Hernández, POMA Study Group

**Affiliations:** 1 Primary Health Care Center Tàrrega, Gerència d'Atenció Primària Lleida, Institut Català de la Salut, Lleida, Spain; 2 CIBER de Diabetes y Enfermedades Metabólicas Asociadas, Instituto de Salud Carlos III, Barcelona, Spain; 3 DAP-Cat group, Unitat de Suport a la Recerca Barcelona, Fundació Institut Universitari d'Investigació en Atenció Primària Jordi Gol i Gurina (IDIAPJGol), Barcelona, Spain; 4 Department of Endocrinology and Nutrition, University Hospital Arnau de Vilanova, Avda. Alcalde Rovira Roure, 80, Lleida, 25198, Spain, +34 973705183; 5 Endocrinology Department, Vall d’Hebron University Hospital, Autonomous University Barcelona, Barcelona, Spain; 6 Diabetes and Metabolism Research Group, Vall d'Hebron Research Institute (VHIR), Barcelona, Spain; 7 Primary Health Care Center, Gerència d'Àmbit d'Atenció Primària Barcelona Ciutat, Institut Català de la Salut, Barcelona, Spain; 8 Research Group in Endocrinology, Diabetes and Nutrition, Institut de Recerca Sant Pau, Barcelona, Spain; 9 Department of Endocrinology and Nutrition, Hospital de la Santa Creu i Sant Pau, Barcelona, Spain; 10 Faculty of Medicine, University of Vic - Central University of Catalonia (UVIC/UCC), Vic, Spain; 11 Institut de Recerca Biomèdica de Lleida Fundació Dr. Pifarré, University of Lleida, Lleida, Spain; 12 Primary Health Care Center, Gerència d'Atenció Primària Lleida, Institut Català de la Salut, Lleida, Spain

 In “Implementation of a Personalized Medicine Approach in Patients With Type 2 Diabetes Mellitus Receiving Multiple Daily Insulin Injections (POMA Project): Protocol for a Before-and-After Intervention Study” [1], the authors made three corrections.

At the beginning of the Acknowledgements section, the following group author contributor information was added:

*The POMA Study Group members are as follows: Magda Mateu Amorós, Anaïs Arqué Badia, Núria Balsells Bailón, Marta Bueno Díez, Marta Carrasco Marín-Blázquez, Alba Dalmau Vila, Carolina López Cano, Raquel Ruano Esteban, Ana Gloria Soler Beunza, Javier Suárez Balaguer, Gabriela Monroy Rodríguez, Josep León Mengíbar, Ferran Rius Riu, Maribel Martí Sotus, Mª José Rosauro Gómez, Laia Naté López, Josefa Tomàs Anastasi, Rocío Quintillà Pueo, Sara Areny Rodrigo, Fernando Chilangua Sepúlveda, Mercè Oliver García, Ruben Perales Sánchez, Anna Serra Peruchet, Jaume Martí Montesa, Irene Gómez Companys, Janette Sandrà Medina, Silvia Guiu Vendrell, Roxana Lenuta Mocanu, David José De la Rica Escuin, Mª Teresa Guasch Clapés, Eva Ribalta Calvet, Rosa M Aran Padulles, Encarna López Esteve, Meritxell Cunillera Batlle, Laura Camats Pascual, Esther Call Camps, Laura García Esteve, Teresa Ibañez Plana, Laia Llubes Arria, Irene Muñoz del Campo, Agustí Jové Subirada, Silvia Tierz Puyuelo, Teresa Vidal Bertran, Maria Mesalles Rodríguez, Natalia Reñé Puigfel, Asunción Garrido Díaz, Sandra Maria Millera, Mª Antònia Lafarga Giribets, Marta Ferminán Miró, Mònica Solanes Cabús, Mª Abel Cucurull Llobet, Laura Roure Chiquero, Salvador Salanova Montané, Mª Fe Diaz Fernandez, Arantxa Morales Valle, Cristina Cebrián Aiguadé, Anna Pagès Cónsul, Neus Miró Vallvé, Raquel Rodríguez Aguilar, Maite Puig Solé, Montserrat Torra Solé, Úrsula Belén Flix Pau, Sara Porta Acosta, and Marta Tolosa Fortuny*.

At the end of the Acknowledgments section, the following sentences were added:

*The authors also acknowledge that the Diabetes Treatment Satisfaction Questionnaire* (*DTSQ*) *is a copyrighted instrument developed by Professor Clare Bradley. Permission to use the DTSQ was obtained from Health Psychology Research Ltd* (*HPR*)*, UK*.

In addition, reference 23 was originally published as:

Gomis R, Herrera-Pombo JL, Calderón A, Rubio-Terrés C, Sarasa P. *RETRACTED ARTICLE: Validación del cuestionario “Diabetes Treatment Satisfaction Questionnaire” (DTSQ) en la población española*. Pharmacoecon Span Res Artic. 2006 Jan;3(1):7–18. doi:10.1007/BF03320906.

This has been replaced with the following:

*Bradley C. Diabetes Treatment Satisfaction Questionnaire: (DTSQ). In: Bradley C, editor. Handbook of Psychology and Diabetes: A Guide to Psychological Measurement in Diabetes Research and Practice. Routledge; 1994. ISBN: 9783718655625*

The correction will appear in the online version of the paper on the JMIR Publications website, together with the publication of this correction notice. Because this was made after submission to PubMed, PubMed Central, and other full-text repositories, the corrected article has also been resubmitted to those repositories.
