# Supplementary material for: Implementation of a Personalized Medicine Approach in Patients With Type 2 Diabetes Mellitus Receiving Multiple Daily Insulin Injections (POMA Project): Protocol for a Before-and-After Intervention Study
Source: JMIR Res Protoc. 2026 Feb 24;15:e85375. doi: 10.2196/85375 (PMC12931837; doi:10.2196/85375)
Supplement: Multimedia Appendix 1 [file resprot-v15-e85375-s001.docx]

## Multimedia Appendix 1. List of study participating centers

| **Primary Health Care Center** (PHCC) |
| --- |
| Agramunt PHCC |
| Alfarràs - Almenar PHCC |
| Guissona – La Segarra PHCC |
| Balaguer PHCC |
| Almacelles PHCC |
| Artesa de Segre PHCC |
| Bellpuig PHCC |
| La Granadella PHCC |
| Les Borges Blanques PHCC |
| Centre Històric-Rambla Ferran PHCC |
| Eixample PHCC |
| Balàfia - Pardinyes - Secà de Sant Pere PHCC |
| Cappont PHCC |
| Bordeta - Magraners PHCC |
| Onze de Setembre PHCC |
| Lleida Rural 1 – Nord PHCC |
| Lleida Rural 2 – Sud PHCC |
| Pla d'Urgell PHCC |
| Ponts PHCC |
| Seròs PHCC |
| Tàrrega PHCC |
