# Supplementary material for: Implementation of a Personalized Medicine Approach in Patients With Type 2 Diabetes Mellitus Receiving Multiple Daily Insulin Injections (POMA Project): Protocol for a Before-and-After Intervention Study
Source: JMIR Res Protoc. 2026 Feb 24;15:e85375. doi: 10.2196/85375 (PMC12931837; doi:10.2196/85375)
Supplement: Multimedia Appendix 2 [file resprot-v15-e85375-s002.docx]

## **Multimedia Appendix 3. Study variables**

| **Socio-demographic variables** | | |
| --- | --- | --- |
| **Variable name:** | **Source** | **Definition, (Value)** |
| Age | eCAP* | Principal, (year of birth) |
| Gender | eCAP* | Others (man, woman, other) |
| Primary Care Center | eCAP* | Others |
| Educational level | Medical history* | Others (Illiteracy, No primary education, Primary education, Secondary education, University studies) |
| Main activity of the company | Medical history* | Others (Student, stable job with fixed hours, stable job with variable hours, intermittent or seasonal work, home care, unemployed, retired/pensioner) |
| Number of children | Medical history* | Other (Number) |
| Date of menopause | Medical history* | Others (year) |
| Sole caregiver | Medical history* | Others (yes, no) |
| **Toxic habits** | | |
| Tobacco consumption | eCAP** | Others (Smoker, non-smoker, ex-smoker) |
| **Comorbidities** | | |
| Time of evolution of diabetes | eCAP* | Main (year of diagnosis) |
| Peripheral arteriopathy | eCAP** | Others (CIE10: I70.xx, I73.xx) |
| Heart failure | eCAP** | Others (CIE10: I50.xx) |
| NYHA | eCAP** | Others (VVK4031) |
| LVEF | eCAP** | Others (VEK402_) |
| Ischemic heart disease | eCAP** | Others (CIE10: I20.xx, I25.xx) |
| Stroke | eCAP** | Others (CIE10: G45.xx, G46.xx, I63.xx, I69.xx ) |
| Chronic kidney disease | eCAP** | Others (ICD10: N18. XX, N19) |
| Diabetic nephropathy | eCAP** | Others (CIE10: E11.2, N08.3, R80) |
| Diabetic neuropathy | eCAP** | Others (CIE10: E11.4, G59, G56, G62, G63, G90, G99) |
| Diabetic retinopathy | eCAP** | Others (CIE10: E11.4, G59, G56, G62, G63, G90, G99) |
| Cardiovascular event | eCAP** | Others (ICD10: I63.9, G45.9, I21.xx) |
| Causes of Mortality | eCAP/INE** | Others (ICD10: R99), specific causes |
| Hospital admission | eCAP** | Other (Number) |
| Cause of hospital admission | eCAP* | Others (CIE10: xx.xx) |
| Severe hypoglycaemia | eCAP** | Other (Number) |
| Dyslipidemia | eCAP** | Others (CIE10: E78.xx) |
| Hypertension | eCAP** | Others (CIE10: I10.xx, I12.xx, I13.xx, I15.xx) |
| **Exploration variables** | | |
| Body mass index | eCAP** | Others (TT103 [kgs/m2]) |
| Abdominal perimeter | eCAP** | Others (EL401-[cm]) |
| Weight | eCAP** | Others (TT102 -[kgs]) |
| Height | eCAP** | Others (TT101 -[cm]) |
| **Clinical variables** | | |
| GADAb | eCAP* | Main |
| C-peptide | eCAP* | Main |
| eGFR (CKD-EPI) | eCAP** | Others |
| Glycohemoglobin (a1c) -: Glycosylated hemoglobin / HbA1c | eCAP** | Main, [fr.subst.] |
| Glucose - Serum Glucose/Glycemia/Glycemia | eCAP** | Others [c.subst. (serum)] |
| HOMA2-IR  HOMA2-ß | Homa calculator | Main |
| **Variables on physical activity, therapeutic adherence, and quality of life** | | |
| IPAQ | Questionnaire** | Seven questions related to physical activity |
| ARMS-E | Questionnaire** | Twelve questions related to adherence to pharmacological treatment |
| EsDQOL | Questionnaire** | Forty-six questions related to the quality of life of patients with diabetes |
| DTSQ-s and DTSQ-c | Questionnaire** | Questionnaire on satisfaction with diabetes treatment |
| **Initial antidiabetic treatment** | | |
| Antidiabetic drugs | eCAP* | Others (ATC/DDD: A10, active ingredient and dosage) |
| **CGM variables** | | |
| Time with the sensor active | AGP Report** | % |
| Time in Row (TIR) | AGP Report** | Time with glucose 70-180mg/dl (%) |
| Low-Range Time (TBR) | AGP Report** | Time with glucose 54-70mg/dl (%) |
| Time in very low range | AGP Report** | Time with glucose <54mg/dl (%) |
| Time in high rank | AGP Report** | Time with glucose 180-250mg/dl (%) |
| Time in very high range | AGP Report** | Time with glucose >250mg/dl (%) |
| Time in Narrow Range | AGP Report** | Time with glucose 70-140mg/dl (%) |
| Coefficient of variability | AGP Report** | % |
| Average glucose | AGP Report** | mg/dl |
| Glucose Control Indicator (GMI) | AGP Report** | % |
| Security | | |
| Adverse events | Ask the participants directly / ecap | Mild/moderate/severe |

*** Baseline, **Baseline and Follow-up**

eCAP: Electronic Clinical Records Software for Primary Care; NYHA: New York Heart Association; LVEF: Left Ventricular Ejection Fraction; GADAb: Glutamic Acid Decarboxylase Autoantibody; eGFR: estimated glomerular filtration rate; CKD-EPI: Chronic Kidney Disease Epidemiology Collaboration; HOMA-B: Homeostatic Model Assessment of Beta Cell Function; HOMA-IR: Homeostatic Model Assessment for Insulin Resistance; IPAQ: International Physical Activity Questionnaire; Adherence to Refills and Medications Scale – European Version; EsDQOL: Spanish Diabetes Quality of Life questionnaire; DTSQ: Diabetes Treatment Satisfaction Questionnaire (status and change version); AGP: Ambulatory Glucose Profile.
